# The therapeutic potential of stem cell-derived exosomes in the ulcerative colitis and colorectal cancer

**DOI:** 10.1186/s13287-022-02811-5

**Published:** 2022-04-01

**Authors:** Gang Guo, Zhaobang Tan, Yaping Liu, Feiyu Shi, Junjun She

**Affiliations:** 1 Center for Gut Microbiome Research, Med-X Institute Centre, First Affiliated Hospital of Xi’an Jiao Tong University, Xi’an, 710061 China; 2 Department of Talent Highland, First Affiliated Hospital of Xi’an Jiao Tong University, Xi’an, 710061 China; 3 Department of Digestive Surgery, Xijing Hospital, Air Force Medical University, Xi’an, 710032 China; 4 Department of Gastroenterology, First Affiliated Hospital of Xi’an Jiaotong University, Xi’an, 710061 China; 5 Department of General Surgery, First Affiliated Hospital of Xi’an Jiao Tong University, Xi’an, 710061 China

**Keywords:** Exosome, Colorectal cancer, Mesenchymal stem cells, Ulcerative colitis

## Abstract

**Background:**

Mesenchymal stem cells (MSCs) therapy is a novel treatment strategy for cancer and a wide range of diseases with an excessive immune response such as ulcerative colitis (UC), due to its powerful immunomodulatory properties and its capacity for tissue regeneration and repair. One of the promising therapeutic options can focus on MSC-secreted exosomes (MSC-Exo), which have been identified as a type of paracrine interaction. In light of a wide variety of recent experimental studies, the present review aims to seek the recent research advances of therapies based on the MSC-Exo for treating UC and colorectal cancer (CRC).

**Methods:**

A systematic literature search in MEDLINE, Scopus, and Google Scholar was performed from inception to December 2021 using the terms [(“colorectal cancer” OR “bowel cancer” OR “colon cancer” OR “rectal cancer”) AND (exosome) AND (stem cell) AND (“inflammatory bowel disease” OR “Crohn's disease” OR “colitis”)] in titles and abstracts.

**Findings:**

Exosomes derived from various sources of MSCs, including human umbilical cord-derived MSCs (hUC-MSCs), human adipose-derived MSCs (hAD-MSCs), human bone marrow-derived MSCs (hBM-MSCs), and olfactory ecto-MSCs (OE-MSCs), have shown the protective role against UC and CRC. Exosomes from hUC-MSCs, hBM-MSCs, AD-MSCs, and OE-MSCs have been found to ameliorate the experimental UC through suppressing inflammatory cells including macrophages, Th1/Th17 cells, reducing the expression of proinflammatory cytokines, as well as inducing the anti-inflammatory function of Treg and Th2 cells and enhancing the expression of anti-inflammatory cytokines. In addition, hBM-MSC-Exo and hUC-MSC-Exo containing tumor-suppressive miRs (miR-3940-5p/miR-22-3p/miR‐16‐5p) have been shown to suppress proliferation, migration, and invasion of CRC cells via regulation of RAP2B/PI3K/AKT signaling pathway and ITGA2/ITGA6.

**Key messages:**

The MSC-Exo can exert beneficial effects on UC and CRC through two different mechanisms including modulating immune responses and inducing anti-tumor responses, respectively.

## Introduction

Colorectal cancer (CRC), the second leading cause of cancer-related deaths worldwide, is a malignancy emerging from the colon and/or rectum [1]. Colon cancer accounts for 72% and rectal cancer for 28% of all CRCs [2]. More than 90% of CRC cases are adenocarcinoma that emerges from the glandular epithelial cells of the colon and/or rectum. CRC arises when these epithelial cells acquire the epigenetic or genetic mutations that confer on them an excessive replication and survival. Such hyper-proliferative cells cause a benign adenoma called polyps, which may then progress to invasive carcinoma (adenocarcinoma) and, eventually over decades, metastasize to different distant organs [2]. Symptoms and signs include blood in the stool and from the rectum, changes in bowel habits and movements, abdominal pain and bloating, fatigue, anemia, and unexplained weight loss [2]. A wide range of risk factors is associated with the incidence of CRC, mainly such as sex, age, hereditary mutations, race and ethnicity, environmental factors and lifestyle, diabetes, cystic fibrosis, as well as ulcerative colitis (UC) [2]. Of note, UC is one of the most important high-risk conditions for CRC incidence and progression. CRC is the most common malignancy among UC patients [3]. The results of a meta-analysis indicated that UC elevates the risk of CRC development by 2.4-fold [4].

UC is a major form of inflammatory bowel disease (IBD), characterized by chronic inflammation and ulcers of the colonic mucosa with variable extension from the rectum toward the cecum. The etiology of UC is complicated and the major pathogenic mechanism is the uncontrolled activation of innate and adaptive immune responses. The former is the initial defense guard against pathogenic factors, and the latter is contributed as the principal culprit of disease development [5]. Briefly, pathogenic factors activate CD4^+^ T cells that differentiate into CD4^+^ T-helper (Th) cells, including Th1 and Th17 cells, and induce the generation of pro-inflammatory M1 macrophages or other pro-inflammatory cells. Both Th1 and Th17 cells secrete a wide range of inflammatory cytokines to provoke intestinal epithelial inflammatory cells infiltrate and chronic colitis [5, 6]. Of note, intestinal epithelial inflammation can be repressed by the differentiation of CD4^+^ FoxP3^+^ regulatory T cells (Tregs) and the supplementary of CD4^+^ Th2 cells. TGF-β and IL-10 released by Tregs provide an immunosuppressive microenvironment that accelerates the repair of the colon mucosal lesion [5]. Overall, inflammation leads to the abnormal secretion of growth and inflammatory cytokines and excessive generation of reactive oxygen species (ROS) that in turn may predispose toward carcinogenesis [7].

The first-line therapy of UC initiates with mesalamine (5-aminosalicylic acid), which may also prevent CRC development [8]. If this treatment does not sufficiently affect, corticosteroids, immunosuppressive agents, biologics (such as anti-tumor necrosis factor-alpha (TNF-α) antibodies, anti-integrin antibodies, anti-interleukin (IL) 12–23 antibodies), and/or non-biologic small molecule tofacitinib may be applied [9]. Despite these medications, an appreciable number of patients undergo persistent flares of inflammation or experience a medication-resistant disease with chronic inflammatory action. In such patients, once no “mucosal healing” (full remission) can be successfully accomplished, the chronic inflammation repeatedly damages the mucosa epithelial surface. The epithelial loss has to be compensated by the elevated proliferation of epithelial cells as a repair mechanism which may eventually be uncontrolled and cause CRC. Consequently, the CRC risk is elevated after a long duration of disease, particularly in patients with chronic active disease [7]. Thus, managing the immune responses can facilitate UC treatment and prevent CRC development.

Mesenchymal stem cells (MSCs) therapy is a novel treatment strategy for cancer and a wide range of diseases with an excessive immune response, such as UC, due to the powerful immunomodulation and immunosuppression properties of MSCs and their capacity for tissue regeneration and repair [10]. As discussed in the following subsections, various kinds of MSCs can provide beneficial effects for treating UC and CRC. The therapeutic mechanism of MSCs mainly focuses on cell-to-cell interaction and paracrine activities. One of the potential therapeutic opportunities can focus on MSC-derived exosomes (MSC-Exo), which have been identified as a type of paracrine interaction, exerting strong immunomodulatory effects. Recent studies suggest that the therapeutic impact of MSCs is majorly dependent on exosomes [11]. In light of a wide variety of recent experimental investigations, the present review aims to seek the recent research advances of therapies based on MSC-Exo for UC and CRC. To this end, we firstly in the next subsection describe an overview regarding the therapeutic potential and challenges of MSCs and then in the following sections would discuss the protective and therapeutic impacts of exosomes derived from different sources of MSCs in UC and CRC.

### The MSC therapy

MSCs can be isolated from various tissues such as the adipose tissue, umbilical cord, and bone marrow. These cells keep pluripotency, transforming into chondroblasts, myocytes, osteoblasts, and adipocytes. MSCs have been found to exert an immunosuppressive impact via polarizing anti-inflammatory M2 macrophages and decreasing both neutrophils and dendritic cells through IL-10 and prostaglandin E2 (PGE2) at the wound site [12]. MSCs are also able to decrease the activity of T and B lymphocytes via modulating their differentiation and proliferation [13, 14].

In preclinical studies, treatment with different sources of MSCs, including bone marrow-derived MSCs (BM-MSCs) [15–17], adipose-derived MSCs (AD-MSCs) [17–19], human umbilical cord-derived MSCs (hUC-MSCs) [20], amnion-derived MSCs (AM-MSCs) [21], and tonsil-derived MSCs (TN-MSCs) [22], have shown protective effects against inflammation and chronic colitis and facilitated mucosal permeability reconstruction in animals with experimental IBD (Table 1). MSCs were found to migrate to lymph nodes to provide immunomodulatory and/or immunosuppressive responses and thereby exert therapeutic impacts on IBD [10]. They can also home to the intestinal muscularis to cause amelioration of IBD through the colon tissue repair [10, 23]. There have also been several phases I/II/III clinical trials that showed transplantation of both autologous and allogeneic MSCs could significantly ameliorate IBD (Table 2). The local (NCT01144962) and intravenous (NCT01090817) administration of BM-MSCs showed no severe adverse events and could significantly induce healing of perianal fistulas and decrease local and systemic inflammation and scores of disease activity index (DAI) in IBD patients [24–28]. In multicenter phase I/IIa [29] and phase III clinical trials (NCT01541579 and NCT01915927), human AD-MSCs transplantation safely achieved remission with long-term healing in IBD patients with treatment–refractory complex perianal fistulas [30–35]. In addition, hUC-MSCs administration was also found to alleviate endoscopic indices and inhibit lymphoid infiltration via inducing immunosuppressive responses in patients with refractory IBD [36].

**Table 1:** Preclinical findings of the MSCs therapy in the experimental IBD

Type of MSCs	Animal model of IBD	Infusion method	Outcome	References
BM-MSCs	DSS-induced mouse model	IV injection	The increase in mucosal permeability The restoration of the damaged colon tissue The decrease in oxidative stress in colitis tissue	[15]
	DSS-induced mouse model	IV injection	The prevention and rapid recovery of weight loss The decrease in inflammatory infiltrates The protection on crypt structure damage	[16]
	TNBS-induced guinea pig model	Enema infusion	The decrease in weight loss The decrease in flattening of the mucosa, hemorrhagic sites, loss of goblet cells, and altered presentation of the circular muscle layer The decrease in leukocyte infiltration to the myenteric ganglia	[17]
	TNBS-induced mouse model	IP injection	The increase in the survival rate The decrease in intestinal inflammation The increase in the expression of anti-inflammatory cytokine IL10 The decrease in the secretion of proinflammatory cytokines TNF-α, IL-12, VEGF The repaired mucosal injury	[19]
AD-MSCs	TNBS-induced guinea pig model	Enema infusion	The decrease in weight loss The decrease in flattening of the mucosa, hemorrhagic sites, loss of goblet cells, and altered presentation of the circular muscle layer The decrease in leukocyte infiltration to the myenteric ganglia	[17]
	TNBS-induced rat model	IV injection	The decrease in the inflammation markers The amelioration of UC The repaired mucosal injury	[18]
	TNBS-induced mouse model	IP injection	The increase in the survival rate The decrease in intestinal inflammation The increase in the expression of anti-inflammatory cytokine IL10 The decrease in the secretion of proinflammatory cytokines TNF-α, IL-12, VEGF The repaired mucosal injury	[19]
hUC-MSCs	DSS-induced mouse model	IP injection	The decrease in mucosal destruction and edema in the submucosa The decrease in colon inflammation The increase in the production of cytokine IL-10 in colon tissue The increase in Treg infiltration in the colon tissue The decrease in the production of proinflammatory cytokines IL-6, IFN-γ, and TNF-α in the colon tissue	[20]
AM-MSCs	TNBS-induced rat model	IV injection	The improvement in the endoscopic and histological changes of colitis The decrease in the infiltration of neutrophils and macrophages The decrease in expression levels of TNF-α, CXCL1, and CCL2	[21]
TN-MSCs	DSS-induced mouse model	IP injection	The improvement in survival rates and body weight gain The increase in scores of disease activity index The normalization of the colon length The decrease in the expression levels of IL-1β and IL-6	[22]

*DSS* dextran sulfate sodium, *IV* intravenous, *TNBS* trinitrobenzene sulfonic acid, *IP* intraperitoneal, *IBD* inflammatory bowel disease, *TNF-α* tumor necrosis factor-α, *VEGF* vascular endothelial growth factor, *IFN-γ* interferon-γ

**Table 2:** Clinical finding**s** of the MSCs therapy in IBD patients

MSC type		IBD type	Administration schedule	Number of patients	Follow-up duration	Clinical phase	Outcome	References
BM-MSCs	Autologous	Luminal CD	Two doses of 1–2 × 10^6^ cells/kg, IV injection, 7 days apart	9	6 weeks	Phase I	The decrease in CDAI score in 3 patients after 6 weeks	[26]
		CD	A single dose of 2 × 10^7^, 5 × 10^7^, or 10 × 10^7^ cells/kg, IV injection	12	2 weeks	Phase I	Safe and feasible at the doses of up to 10 million cells/kg	[27]
	Allogeneic	Perianal fistulizing CD	A single dose of 1 × 10^7^, 3 × 10^7^, or 9 × 10^7^, local injection	24	24 weeks	Phase II	No severe adverse events 3 × 10^7^ MSCs induced healing of perianal fistulas	[24]
		UC/CD	A single dose of (1.5–2) × 10^8^, IV injection	21	6, 12, and 24 weeks	Phase II	The decrease in the clinical and morphological indices of inflammatory activity in 34 (72%) patients	[28]
		Luminal CD	A single dose of 2 × 10^6^ cells/kg weekly for 4 weeks, IV injection	16	6 weeks	Phase II	The decrease in CDAI score in 15 patients after 6 weeks	[25]
AD-MSCs	Autologous	Perianal fistulizing CD	2 × 10^7^ cells/kg, local injection	12	24 weeks	Phase I	Complete clinical healing in 9 patients after 12 weeks Complete clinical healing in 10 patients after 24 weeks	[33]
		Perianal fistulizing CD	1 × 10^7^, 2 × 10^7^, or 4 × 10^7^ cells/ml in three groups	9	8 weeks	Phase I	2 patients treated with 2 × 10^7^ cells/ml showed complete healing and 3 patients treated with 4 × 10^7^ cells/ml showed complete healing at week 8	[34]
		Perianal fistulizing CD	3 × 107 cells per centimeter length of the fistula	43	8 weeks	Phase II	Complete fistula healing in 27/33 (82%) patients after 8 weeks Complete closure of fistula in 23/26 (88%) patients after 1 year	[32]
	Allogeneic	Perianal fistulizing CD	A single dose of 1.2 × 10^8^, Intralesional injection	212	24 weeks	Phase III	Combined remission in 50% of patients	[30]
		Perianal fistulizing CD	A single dose intralesionally If 2 × 10^7^ failed, 4 × 10^7^ subsequently	24	24 weeks	Phase II	The decrease in the number of draining fistulas in 69.2% of patients Complete fistula closure treated in 56.3% of patients Complete closure of all existing fistula tracts in 30% of patients	[35]
hUC-MSCs	Allogeneic	UC/CD	1 × 10^6^ cells/kg, IV injection	7	6 months	Phase I	The decrease in clinical activity index scores in all patients The decrease in fistula size and drainage in one patient The decrease in rough mucosa, polypoid lesions, and ulcers in three patients The decrease in the extent of the inflamed area and the dense lymphocytic infiltration in the mucosa propria A relapse in two patients at 6 and 7 months after treatment At the 3-month visit, five patients achieved remission and maintenance of remission lasted for more than 24 months in two patients	[36]

Besides, MSCs have also been found to exert a certain therapeutic impact on CRC. MSCs could prolong patients' survival time who suffered from metastatic tumors and complications caused by CRC [37]. MSCs therapy was also found to prevent CRC development in UC patients via suppressing chronic inflammation, as well as exerting a chemoprophylaxis role [38]. In the tumor microenvironment, MSCs have been found to regulate the immunologic functions by altering the cytokines secreted from immune cells such as T cells [39].

Nevertheless, various problems have been evidenced in clinical trials of MSCs for UC and CRC treatment. Of note, the important limiting challenge is the low therapeutic efficiency of MSCs due to their low survival rate and immunosuppressive potential in the process of migration to the intestinal mucosa [10]. To circumvent these limitations, various valid MSCs engineering approaches have been employed for UC and CRC. These include pretreatments that improve immunomodulation activity and homing. For instance, engineered MSCs expressing high levels of transforming growth factor-β1 (TGF-β1) [40] or interferon-γ (IFN-γ) [41] showed the enhanced immunosuppressive activity leading to effective amelioration of colonic inflammation in colitis mice. In addition, TLR3-preconditioned hUC-MSCs were found to secrete extra immunosuppressive cytokines and inhibit proliferation of active T cells and thus showed a higher therapeutic efficiency, compared with un-preconditioned hUC-MSCs, to ameliorate the clinical and histopathological severity of acute UC in a murine model of IBD [42]. In another study, aspirin (acetylsalicylic acid, ASA)-pretreated BM-MSCs have significantly improved immunomodulatory activity by downregulation of Th17 cells and upregulation of regulatory T cells (Tregs) via the 15d-PGJ2/PPARγ/TGF-β1 pathway [43]. Of note, ASA-pretreated BM-MSCs markedly alleviated colonic inflammation and the DAI score in mice with experimental UC [43]. Some pretreatments have been also used to improve homing of MSCs. Receptor CXC chemokine receptor 4 (CXCR4) plays a key role in chemotaxis and stem cell homing through interaction with its specific ligand stromal cell-derived factor (SDF-1). It was reported that over-expression of CXCR4 could increase in vivo migration and engraftment of MSCs into the inflamed colon where these cells act as an immunomodulatory and/or anti-inflammatory component of the immune system in mice with experimental UC [44].

MSCs have been found to exert their role through the cell-to-cell and paracrine interactions. Paracrine active substances suppress the proliferation and induce the apoptosis of Th1 and Th17 cells and other immune cells so as to prevent excessive immunity. Exosomes, a kind of MSCs paracrine, have also been found to be capable of replacing MSCs in the treatment of UC and CRC in recent years.

### Exosomes

**Fig. 1:**
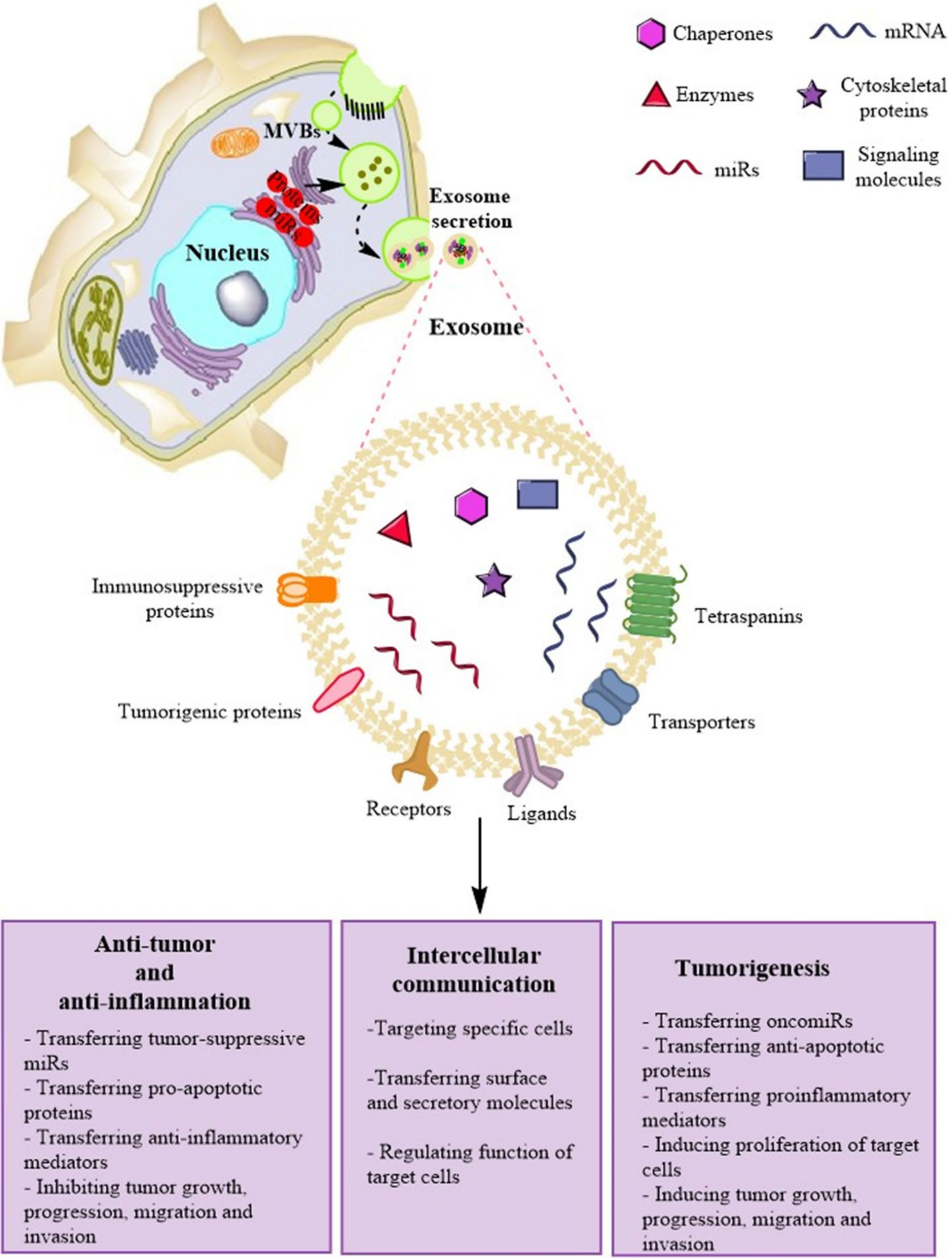
Schematic depicting the biogenesis, structure, and major molecular components of exosomes. Exosomes are lipid bilayer extracellular vesicles with diameters typically between 30 and 120 nm. Exosome biogenesis initiates when a portion of the plasma membrane buds into the cell to create an early endosome that transforms into late endosomes termed multivesicular bodies (MVB) containing a large number of exosomes. In the meantime, transmembrane and surface proteins located on the plasma membrane are placed into the invaginating membrane, while the cytosolic biologics are enveloped within the exosomes. After fusion with the plasma membrane and through the exocytosis, MVBs release the exosomes into the extracellular space. The payload of exosomes can include proteins, cytoskeletal proteins, signaling molecules, receptors, ligand, tetraspanins, miRs, mRNAs, and other bioactive compounds. They can also carry various immunoregulatory (like miRs) and immunosuppressive (like surface proteins) as well as anti-tumor (like miRs) and tumorigenic (like surface proteins) mediators

**Fig. 2:**
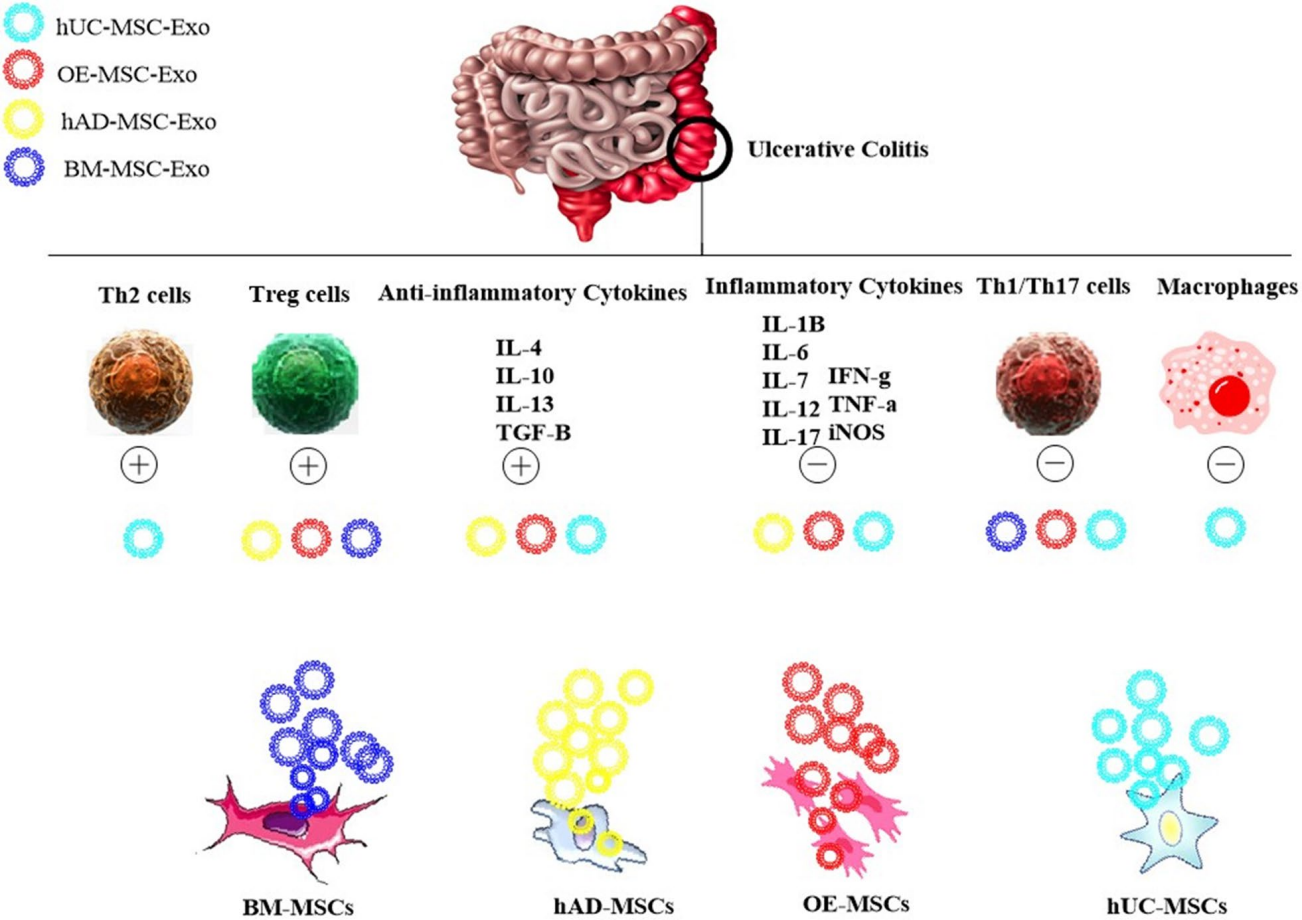
The modulatory effect of MSC-Exo on the dysregulated immune system components in the experimental UC. The hUC-MSC-Exo inhibit inflammatory macrophages while promoting the anti-inflammatory activity of Th2 cells. Over-activated Th1/Th17 cells can be suppressed by hBM-MSC-Exo, hUC-MSC-Exo, and OE-MSC-Exo. The anti-inflammation activity of Treg cells can be improved by hBM-MSC-Exo, hAD-MSC-Exo, and OE-MSC-Exo. Over-expressed proinflammatory cytokines and hUC-MSC-Exo, hAD-MSC-Exo, and OE-MSC-Exo can reduce over-expressed proinflammatory cytokines while increasing the expression of pro-inflammatory cytokines

**Fig. 3:**
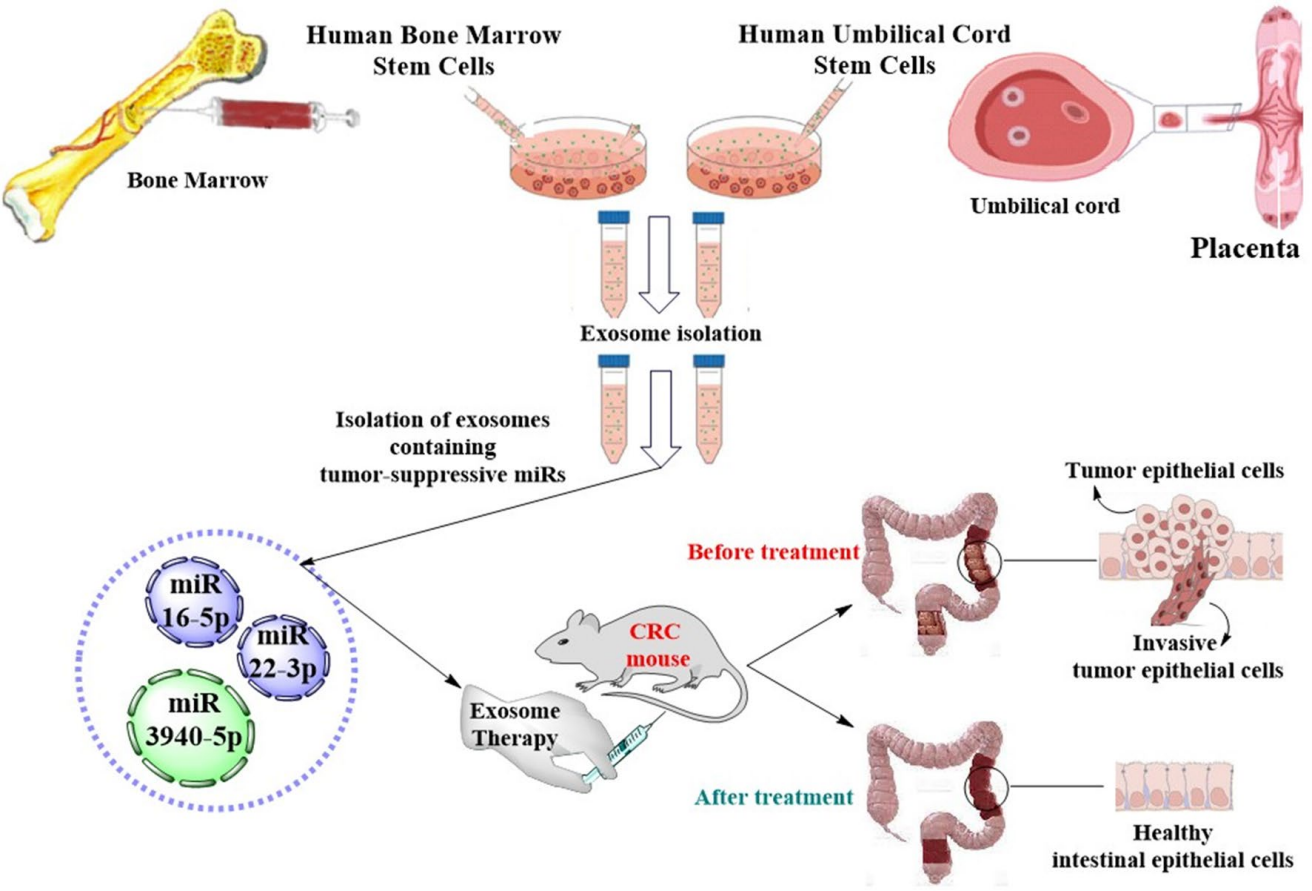
A schematic view of the exosome therapy for CRC treatment using MSC-derived exosomes containing tumor-suppressive miRs

Exosomes are lipid bilayer extracellular vesicles (EVs) secreted by a broad range of cell types such as MSCs. These vesicles mirror the molecular content of donor cells and participate in cellular cross talk between both neighboring and distant cells. Exosome-mediated intercellular communication stems from its capacity in cell-to-cell transferring of biological information. The composition of the exosomes is highly dependent on the parental cells and their biogenesis pathways. This composition determines the function of the exosomes. Some components exist in almost all exosomes, and some are tissue- and cell-specific [45, 46]. In general, the exosomes are enriched in DNA, mRNA, microRNAs (miRs), and bioactive proteins which can mediate the functions of exosomes [47] (Fig. 1). Stem cell-derived exosomes have been demonstrated to not only reiterate the therapeutic properties of origin cells but also donate advantages over them [48]. They show the potential to overcome limitations of stem cell therapy, like restricted engraftment and low survival rate of stem cells, risk of differentiation into undesired cell lines and development of ectopic tissues, risk of tumorigenicity and genetic aberrations, as well as ethical and safety challenges [48]. Notably, exosomes have autonomous targeting capabilities and can home to a specific lesion tissue [48]. Exosomes have been found to internalize in a cell type-specific route that relies on recognition of exosomal surface ligands/receptors by the target cell or tissue. As discussed in the following sections, a growing number of preclinical investigations indicates that exosomes derived from various sources of MSCs could exert anti-inflammatory and immunomodulatory effects on UC (Tables 3, 4 and Fig. 2) and CRC (Table 3 and Fig. 3). Thus, the present review aimed to highlight stem cell-derived exosomes reported as the potential therapeutic tools for treating UC and CRC.

**Table 3:** The biological characteristics of stem cell-derived exosomes in experimental CRC and UC

Source of exosome	Biological characteristics in the CRC model (References)		Biological characteristics in the UC model (References)
UC-MSCs	Tumor-suppressive exosomes	Suppressing invasion and the EMT of CRC cells Inhibiting the tumor growth and metastasis [72]	Homing to the inflammatory site of the colon tissue Inhibiting the macrophages infiltration to the inflamed colon Inhibiting the expression of pro-inflammatory mediators Regulating macrophage pyroptosis Preventing the colon shortening [49–52]
	Tumorigenic Exosomes	NP	
AD-MSCs	Tumor-suppressive exosomes	NP	Homing to the inflammatory site of the colorectal tissue Inhibiting inflammatory cell infiltration and colonic inflammation Preventing alterations of colon length and crypt loss Preventing rectal bleeding Reducing histological scores of DAI [68]
	Tumorigenic Exosomes	NP	
BM-MSCs	Tumor-suppressive exosomes	Suppressing proliferation, migration, and invasion of CRC cells Inducing the apoptosis of CRC cells Repressing the tumor growth and progression Reducing inflammation in the tumor microenvironment [77, 79, 82, 83, 119]	Inhibiting inflammatory cell infiltration Restoring epithelial ulceration and aberrant crypt architecture Reducing histological scores of DAI [70]
	Tumorigenic Exosomes	Enhancing proliferation, migration, and colony formation of CRC cells Inducing progression of CRC Increasing the population of CSCs in CRC cells [96, 97, 101]	
OE-MSCs	Tumor-suppressive exosomes	NP	Increasing the colon length Inhibiting inflammatory cell infiltration and colonic inflammation Reducing colon shortening and crypt loss Reducing histological scores of DAI [71]
	Tumorigenic Exosomes	NP	

*DAI* disease activity index, *EMT* epithelial–mesenchymal transition, *CRC* colorectal cancer, *UC* ulcerative colitis, *CSCs* colon cancer stem cells, *NP* not reported, *MSCs* mesenchymal stem cells, *UC-MSCs* umbilical cord-derived MSCs, *AD-MSCs* adipose-derived MSCs, *BM-MSCs* bone marrow-derived MSCs, *OE-MSCs* olfactory ecto-MSCs

**Table 4:** Therapeutic theory of MSCs-derived exosomes in the experimental UC

Source of exosome	Therapeutic theory	Molecular target	References
UC-MSCs	↓ Macrophage infiltration ↓ IL-1β, IL-6, IL-7, TNF-α, iNOS ↑ IL-10, IP-10	Ubiquitin components Ubiquitin-associated molecules	[49, 50]
	↓ Macrophage pyroptosis	NLRP3	[51]
	↓ Th17 cells	TSG-6	[52]
	↑ Th2 cells	TSG-6	[52]
AD-MSCs	↑ Treg cells ↑ IL‐4, IL‐10, IL-13, TGF‐β ↓ IL-1β, IL-6, IL-12, IL-17, TNF-α, IFN‐γ	Not defined	[68, 69]
BM-MSCs	↑Treg cells ↓Th17 cells	Stat3 inhibition mediated by exosomal miR-125a and miR-125b	[70]
OE-MSCs	↑ Treg cells ↑ TGF-β, IL-10 ↓ Th1/Th17 cells ↓ IL-17, IFN-γ	Not defined	[71]

↓ and ↑ show the decrease and the increase, respectively

*MSCs* mesenchymal stem cells, *UC-MSCs* umbilical cord-derived MSCs, *AD-MSCs* adipose-derived MSCs, *BM-MSCs* bone marrow-derived MSCs, *OE-MSCs* olfactory ecto-MSCs, *TNF-α* tumor necrosis factor-α, *TSG-6* TNF-α stimulated gene 6, *NLRP3* NOD-like receptor family, pyrin domain-containing 3

## The search strategy

A systematic literature search was performed in MEDLINE (http://www.ncbi.nlm.nih.gov/pubmed), Scopus (http://www.scopus.com), and Google Scholar (http://scholar.google.com), without any language restrictions, to identify all published articles dealing with the aims of the present study. The search was performed from inception to December 2021 using the terms [(“colorectal cancer” OR “bowel cancer” OR “colon cancer” OR “rectal cancer”) AND (exosome) AND (stem cell) AND (“inflammatory bowel disease” OR “Crohn's disease” OR “colitis”)] in titles and abstracts.

## The therapeutic potential of MSC-derived exosomes in UC treatment

### Exosomes from human UC-MSCs

hUC-MSCs have been found to play a key role in the formation of tissue regeneration from inflammation and tissue damage. There is growing preclinical evidence that indicates exosomes derived from hUC-MSCs (hUC-MSC-Exo) can mediate therapeutic effects of hUC-MSCs and protect against tissue damage in UC [49–52].

It was shown that intravenously injected hUC-MSC-Exo can home to the colon tissue and, subsequently, increase the survival rate and alleviate the severity of UC symptoms, including the colon shortening, the destroyed integrity of colon structure, and the increased infiltration of inflammatory cells in colitis mice by regulating the inflammatory responses [49, 50]. The infiltration of inflammatory cells, particularly macrophages, is a hallmark feature of UC. Inflammatory M1 macrophages can secrete various proinflammatory cytokines and enzymes, which would lead to the damage of colon tissue. Of note, it was shown that the recruitment of macrophages to the inflamed colon and the expression of pro-inflammatory mediators, including IL-1β, IL-6, IL-7, TNF-α, and iNOS, were significantly decreased in hUC-MSC-Exo-administrated colitis mice, while the expression of anti-inflammatory mediators, such as IL-10 and IP-10, was significantly increased [49, 50]. Notably, the hUC-MSC-Exo were found to ameliorate the inflammation and UC symptoms through regulating the modification of ubiquitination [49]. Ubiquitination, a process by which the target protein is modified by ubiquitin molecules, plays a regulatory role in numerous biological functions such as inflammatory responses in UC [53, 54]. The ubiquitin-associated molecules such as K48, K63, and FK2 are known to promote inflammation through activating NF-κB and mTOR signaling pathways, and hUC-MSC-Exo administration markedly decreased the protein expression of these molecules in the colon tissue of colitis mice [49]. Further, it was shown that the gene expression levels of ubiquitin-like modifier activating enzyme 3 (Uba3), ubiquitin-conjugating enzyme E2M (UBE2M), and NEDD8 activating enzyme E1 (NAe1) as the central components of the ubiquitination were profoundly decreased in hUC-MSC-Exo-administrated mice, supporting the role of ubiquitin modification in anti-inflammatory effects of hUC-MSC-Exo in UC [49].

On the other hand, macrophage pyroptosis, a process of cell death through induction of NLRP3 (NOD-like receptor family, pyrin domain-containing 3) inflammasomes, has been found to partially account for inflammatory responses in colitis. There have also been experimental studies that showed hUC-MSC-Exo alleviated UC by regulating macrophage pyroptosis via the miR-378a-5p/NLRP3 axis [51]. Of note, the in vivo study indicated that intravenous administration of hUC-MSC-Exo containing high levels of miR-378a-5p could suppress the expression of NLRP3 inflammasome-related molecules, including pattern recognition receptor NLRP3, apoptosis-associated speck-like protein containing a caspase recruitment domain (ASC), caspase-1, IL-1β, and IL-18, in colitis mice. This resulted in the reduction in macrophage pyroptosis in the mouse colon and protection against colitis [51]. The same result was found through the in vitro cell experiments, where the co-culture of hUC-MSC-Exo with human myeloid leukemia mononuclear (THP-1) cells and mouse peritoneal macrophages (MPMs) caused the reduced expression of NLRP3 inflammasomes and the elevated cell survival [51].

Another in vivo study showed that intraperitoneal administration of hUC-MSC-Exo ameliorated UC symptoms and decreased the mortality rate in colitis mice. It was found that hUC-MSC-Exo protect against UC through restoring the intestinal function and intestinal immune homeostasis via TNF-α-stimulated gene 6 (TSG-6), a glycoprotein with the capacities of tissue repair and immune regulation [52]. The intestinal barrier, which is disrupted in UC, includes tight junctions (TJs) from the intestinal epithelial cells (IECs) and the mucus layer [55]. The TJ proteins, mainly including claudin-1, occludin, and ZO-1, are essential for maintaining the integrity and function of the intestinal barrier [56]. Mucus is generated and released by goblet cells in the IECs [56], and mucosal permeability will be elevated after the disruption of the intestinal barrier [57]. Of note, the protective effect of hUC-MSC-Exo on the intestinal barrier was substantiated by the increased number of goblet cells and reduced permeability of intestinal mucosa. Such an alleviating effect was accompanied by improving the impaired structure of TJ and microvilli, as well as elevating the expression of TJ proteins in the colon tissue [52]. In the case of intestinal inflammation, microarray analysis indicated that hUC-MSC-Exo treatment upregulated the expression of the anti-inflammatory cytokines, including chemokine ligand 14 (CXCL14), IL-1β, IL-11, and IL-12 and downregulated the pro-inflammatory cytokines, including IL-4 and TGF-β, in the colonic mucosal [52]. Moreover, the imbalance of T cell subtypes has a key role in the onset and progression of UC [58]. Notably, hUC-MSC-Exo also elevated the anti-inflammatory response of Th2 cells and decreased the pro-inflammatory response of Th17 cells in the mesenteric lymph nodes [52]. Mechanistically, TSG-6 was identified to be one of the key mediators of the therapeutic activity of hUC-MSC-Exo in UC [52]. It can be further supported by other investigations that showed the therapeutic effect of stem cells in IBD animals is majorly mediated via TSG-6 [59–61]. Indeed, TSG-6 is a secreted protein that has tissue-protective and anti-inflammatory features [62, 63]; after sensing the inflammatory signal, the corresponding cell secretes TSG-6 to the injury site to inhibit immune response and repair the injured tissue [64, 65].

### Exosomes from human AD-MSCs

Exosomes derived from human AD-MSC (hAD-MSC-Exo) have been reported to efficiently repair damaged tissues, such as adipose tissue inflammation [66] and cutaneous wounds [67]. A recent experimental study revealed that hAD-MSC-Exo can also exert a protective role against UC in experimental colitis. It was reported that intravenously injected hAD-MSC-Exo could home to the inflammatory site of the colorectal tissue and, subsequently, attenuate the severity of UC in colitis mice [68]. Of note, the typical symptoms of UC, such as the loss of body weight, alterations of colon length, colonic inflammation, and rectal bleeding, were dramatically attenuated by hAD-MSC-Exo administration [68]. hAD-MSC-Exo-administrated mice maintained relative integrity of colon structure with no apparent ulceration and showed less inflammatory cell infiltration and less crypt loss, as well as lower histological scores of DAI during the disease progression [68]. hAD-MSC-Exo also increased levels of anti-inflammatory cytokines (IL-10 and IL-13) and decreased the pro-inflammatory cytokines (IL-1β, IL-6, IL-12, TNF-α) in both colon tissue and peripheral blood serum [68]. Notably, hAD-MSC-Exo maintained the colonic integrity by reshaping the cell structure in colon mucosa via inducing the regeneration and proliferation of intestinal stem cells, epithelial cells, and goblet cells in colon crypts [68]. This finding is in agreement with the report of another study that indicated the intraperitoneal injection of exosomes derived from mouse AD-MSC (mAD-MSC-Exo) can mediate a significant immunomodulatory effect and, consequently, attenuate the DAI score and the severity of symptoms including bleeding, colon shortening, colon injury, and body weight loss in mice with acute colitis [69]. Of note, mAD-MSC-Exo administration could decrease the levels of pro-inflammatory cytokines, including IFN‐γ, TNF‐α, IL‐12, and IL‐17, and increase percentages of anti-inflammatory Treg cells and the anti-inflammatory cytokines including IL‐4, IL‐10, and TGF‐β in lymph node and spleen of colitis mice [69].

### Exosomes from BM-MSCs

Some findings show that IFN-γ treatment can efficiently improve the therapeutic potential of MSCs against colitis via enhancing their immunomodulation activity by affecting MSC-secreted exosomes. A recent study reported that the systemic infusion of exosomes derived from BM-MSCs (BM-MSC-Exo) could significantly ameliorate body weight loss, colon shortening, and the DAI score of colitis and reduce the ratio of the Th17 cells with an increased ratio of Treg cells in colitis mice [70]. Interestingly, administration of exosomes derived from IFN-γ-primed BM-MSC revealed superior improving impacts to colitis [70]. Histologically, the intestinal structure, which was impaired with epithelial ulceration, aberrant crypt architecture, and inflammatory cell infiltration, was restored more efficiently in colitis mice treated with IFN-γ-primed BM-MSC-Exo than non-primed ones [70]. It was found that the therapeutic effects of exosomes on colitis are partly mediated by the reciprocal regulation of Th17 and Treg cells. Mechanistically, the exosome treatment was found to significantly decrease the Stat3 expression to inhibit differentiation of Th17 cells, which also might result in an increased number of Treg cells [70]. Of note, exosomal miR-125a and miR-125b were found to directly target and inhibit Stat3 and consequently repress the Th17 differentiation, whereby resulted in attenuating symptoms of colitis in exosome-administrated mice. Notably, IFN-γ pretreatment could intensify the capacity of BM-MSC-Exo to ameliorate colitis phenotypes in the mouse model by upregulation of miR-125a and miR-125b expression [70].

### Exosomes from olfactory ecto-MSCs

Olfactory ecto-MSCs (OE-MSCs) are a novel population of resident stem cells in the olfactory lamina propria with considerable immunosuppressive activity. In colitis mice, the intravenous administration of exosomes derived from OE-MSCs (OE-MSC-Exo) markedly increased the colon length, reduced the DAI score and inflammation, and retained the integral structure of the colon, and with less lymphocytic infiltration, decreased the crypt loss and lowered the histopathological scores [71]. The ameliorative effect of OE-MSC-Exo on the disease severity was accompanied by diminished Th1/Th17 cell pro-inflammatory responses and enhanced Treg cell anti-inflammatory responses [71]. Mechanistically, OE-MSC-Exo administration was found to exert immunomodulatory effects by suppressing differentiation of Th1/Th17 cells and inducing the differentiation of Treg cells as well as inhibiting T cell proliferation via reducing IL-17 and IFN-γ and enhancing TGF-β and IL-10 production by the T cells [71].

## The therapeutic potential of MSC-Exo in the CRC treatment

Several investigations have evaluated the effects of MSCs on CRC; however, the results are controversial. Some investigations have shown the inhibitory effect of MSCs on cancer cell proliferation and migration, while others reported the supportive effects (Table 5 and Fig. 3). There is evidence that shows the dual function of MSCs on cancer cells is due to their secretome, mainly exosomes. Indeed, it has been found that the cargo content of MSC-derived exosomes (MSC-Exo) can majorly determine the effects of MSCs on cancer cells. Of note, MSC-Exo containing anti-tumor mediators can inhibit the tumor growth and, thus, show potentiality as a therapeutic tool, while those contacting tumorigenic mediators can promote the tumor growth and, thus, have potential as the therapeutic target in cancer treatment. On the other hand, MSC-Exo, due to their lipid bilayer membrane, provide a suitable delivery vector that can protect therapeutic molecules from degradation. Therefore, potential MSC-Exo-based therapeutic approaches can be categorized into three groups including using tumor suppressor MSC-Exo as therapeutic tools, suppressing tumorigenic MSC-Exo as the therapeutic target, and using MSC-Exo as therapeutic carriers. In the following sections, we discuss such MSC-Exo-based therapeutic approaches in the CRC treatment.

**Table 5:** Therapeutic MSCs-derived Exo-miRs in CRC treatment

Source of exosome	Exo-miR	Molecular target	References
*Tumor-suppressive MSC-Exo-miRs*			
hUC-MSCs	miR-3940-5p	ITGA6	[72]
hBM-MSCs	miR-22-3p	RAP2B/PI3K/AKT pathway	[82]
hBM-MSCs	miR‐16‐5p	ITGA2	[83]
*Tumorigenic MSC-Exo-miRs*			
hBM-MSCs	miR-424	TGFBR3	[97]
hBM-MSCs	miR-142-3p	Inhibiting the Numb expression and promoting the Notch signaling pathway	[101]
hCC-MSCs	miR-30a miR-222	MIA3	[102]

*ITGA6* integrin alpha 6, *ITGA2* integrin alpha 2, *CRC* colorectal cancer, *MSCs* mesenchymal stem cells, *Exo-miRs* exosomal microRNAs, *MSC-Exo-miRs* MSC-derived Exo-miRs, *hCC-MSCs* human CRC-derived MSCs, *hUC-MSCs* human umbilical cord-derived MSCs, *hBM-MSCs* human bone marrow-derived MSCs, *MIA3* melanoma inhibitory activity protein 3, *TGFBR3* transforming growth factor beta receptor 3

### MSC-Exo as potential therapeutic tools for the CRC treatment

There is growing evidence that shows exosomes isolated from different sources of MSCs can exert a suppressive effect on CRC progression. It was reported that hUC-MSCs administration could promote the differentiation of Treg cells by activating Smad2 signaling so as to suppress the colitis and inhibit the development of colitis-associated CRC [38]. Further studies indicated that exosomes from hUC-MSCs delivered high levels of miR-3940-5p into CRC cells which directly downregulated the integrin alpha 6 (ITGA6) expression, by which suppressing the invasion and epithelial–mesenchymal transition (EMT) of CRC cells in vitro and inhibiting the tumor growth and metastasis in vivo [72]. Mechanistically, ITGA6 expression has been found to involve in cancer progression and metastasis [73–75] by regulating the tumor microenvironment and maintaining tumor cells [76]. Other reports showed that BM-MSCs could significantly ameliorate the colitis-associated CRC and then lengthen the colon and relieve weight loss in the IBD mice by inhibiting activation of STAT3 (signal transducer and activator of transcription 3) and the expression of pro-inflammatory cytokines such as TNF-α, IL-1, and IL-6 in the colon tissue [77, 78]. The suppressive effect of BM-MSCs on inflammation-mediated tumorigenesis in the colon was supported by another study that demonstrated that BM-MSCs administration could inhibit the progression of CRC in rat models by modulating immune components and reducing the immune-related inflammation in the tumor microenvironment [79]. Notably, BM-MSC-Exo have been found to be rich in miR-22-3p which is a tumor suppressor and represses the proliferation and progression of tumors. miR-22-3p was shown to downregulate the expression levels of specificity protein 1 (Sp1) [80] and astrocyte-elevated gene-1(AEG-1) [81], consequently suppressing the tumor cell proliferation in hepatocellular carcinoma [80] and non-small cell lung cancer (NSCLC) [81]. Of note, it was reported that Exo-miR-22-3p derived from human BM-MSCs (hBM-MSCs) could suppress CRC cell proliferation and invasion by regulating RAP2B/PI3K/AKT pathway in SW480 cancer cell line [82]. In this line, another in vitro study reported that BM-MSC-Exo containing a high level of miR‐16‐5p suppressed proliferation, migration, and invasion, while simultaneously inducing the apoptosis of the CRC cells via downregulation of ITGA2 [83]. It was further supported by in vivo experiments that showed the administration of BM-MSCs‐Exo overexpressing miR‐16‐5p could decrease the expression of ITGA2 in tumor tissues and, thereby, significantly reduce the volume and growth ability of CRC tumor. Notably, the tumor-suppressive activity of miR‐16‐5p has been already confirmed by other studies that showed the miR‐16‐5p expression was downregulated, while the expression of its oncogenic targets, ITGA2 and Smad3, was upregulated in various cancer cells such as gastric cancer [84, 85], pancreatic ductal adenocarcinoma [86], chordomas [87], and CRC [83], which was contributed to an unfavorable survival rate. Taken together, these findings suggest that therapeutic effects of MSCs on the CRC may be attributable to secreted exosomes enriched in tumor-suppressive miRs (Table 5). Notably, the exosome therapy using hUC-MSC-Exo containing miR-3940-5p and BM-MSC-Exo containing miR-22-3p and miR‐16‐5p, or elevating the CRC expression of such tumor-suppressive miRs can be a promising approach for treating patients with CRC in the future clinical practices.

### MSC-Exo as potential therapeutic targets for the CRC treatment

Despite evidence for inhibitory effects of MSCs and MSC-Exo, there are also reports that show their promoting effects in the progression of CRC in animal models, suggesting them as potential therapeutic targets that their inhibition can provide a valuable approach in the treatment of CRC. MSCs have been found to exert tumorigenic activities on CRC through various mechanisms including inhibiting the cell apoptosis and promoting cell proliferation, migration, and invasion [88, 89] as well as inducing angiogenesis [90–92] and drug resistance [93, 94].

There is growing evidence showing that MSC secretome, particularly exosomes, mediates the supporting role of MSCs in cancer cell growth and metastasis [95]. An in vitro study of CRC cell lines cultured in hBM-MSCs conditioned media indicated that the secretory fraction of hBM-MSCs strongly induced the progression of the cancer cells by enhancing cell proliferation, migration, and colony formation through AMPK/mTOR-mediated NF-κB activation [96]. A recent in vitro study reported that exosomes secreted from hBM-MSCs could induce CRC development through transporting miR-424 into colon cells [97]. The Exo-miR-424 was found to accelerate the progression of CRC by targeting the tumor suppressor receptor TGFBR3 [97]. Of note, miR-424 has been already known as a tumorigenic miR that is highly overexpressed in several human cancer cells including NSCLC [98], tongue squamous cell carcinoma (TSCC) [99], and also CRC [95, 100]. It was shown that miR-424 was upregulated, while TGFBR3 was downregulated in CRC tissues and cell lines [95]. Importantly, the expression of miR-424 and TGFBR3 was also found to be correlated with tumor differentiation degree, tumor infiltration depth, TNM (tumor, node, and metastasis) stage, vascular invasion, as well as lymphatic node metastasis (LNM), and distant metastasis in CRC patients [97]. Notably, it was shown that inhibition of miR-424 could limit the development of CRC via overexpressing TGFBR3 [95]. The inhibition of miR-424 and overexpression of TGFBR3 could suppress migration and invasion of CRC cells, arrest the CRC cells at G0/G1 phase, and induce CRC cell apoptosis, while BMSC-Exo exerted an inverse effect [97]. Furthermore, it was also reported that BM-MSC-Exo containing miR-142-3p significantly increase the population of colon cancer stem cells (CSCs) in CRC cells [101]. Of note, depriving miR-142-3p from BM-MSC-Exo clearly reduced the population of CSCs [101]. Mechanistically, Numb, a suppressor of the Notch signaling pathway, was identified to be the target gene of miR-142-3p. Indeed, miR-142-3p downregulates the Numb expression and, thereby, induces the expression of Notch target genes, such as Cyclin D3, P21, and Hes1, resulting in promoting the CSC phenotype [101]. Collectively, these findings indicate hBM-MSC-Exo-miR-424 and Exo-miR-142-3p as the promising therapeutic targets (Table 5) whose inhibition may provide a novel strategy in CRC treatment. Notably, inhibiting tumorigenic miR-424 and miR-142-3p in exosomes from hBM-MSCs may suppress tumorigenic activity of such exosomes and restore therapeutic activity of hBM-MSC-Exo mentioned in the last section,

In addition, another study reported that exosomes from human CRC-derived MSCs (hCC-MSC-Exo) could constitute a key cell-to-cell cross talk in tumor stroma that supports tumor growth and progression (Table 4) [102]. Screening the miR profiles indicated that the levels of oncogenic miR-30a and miR-222 are markedly enriched in hCC-MSC-Exo [102]. Of note, it was shown that Exo-miR-30a and Exo-miR-222 secreted from hCC-MSCs are efficiently delivered into the CRCs [102] where directly target tumor-suppressive protein MIA3 and downregulate its expression to induce the ability of colon cells to proliferate, migrate, and metastasize [102, 103]. Importantly, suppressing both miR-30a and miR-222 could reverse the tumor promotion effect of hCC-MSC-Exo in colon cancer cells [102]. To sum up, these findings suggest that the blockade of the exosomal transfer of tumorigenic miR-30a and miR-222 by hCC-MSCs can provide a promising approach to inhibit the progression of CRC.

A useful tool to efficiently knock down the function of a specific miR in cells or exosomes is direct transfection of chemically synthesized miR inhibitors, commonly referred to as miR decoys, miR sponges, AMOs (anti-miRNA antisense oligonucleotides), and antagomirs or anti-miRs. Generally, these are antisense molecules that, through complementary sequences, bind and sequester specific miRs from their mRNA targets. Miravirsen, a specific inhibitor of miR-122, is the major example and first miR inhibitor AMO drug that entered clinical trials for treating hepatitis C [104].

### MSC-Exo as therapeutic carriers for the CRC treatment

Exosomes hold great potential as the carrier of therapeutic agents for the tumor-targeted therapy [105–111], due to their low immunogenicity, high biocompatibility and stability, high encapsulation capacity, and facilitating internalization of the cargo into tumor cells. Moreover, exosomes are able to be easily decorated with particular ligands on their surface to improve targeting to the tumor microenvironment [112–114].

Genetic materials, such as anti-tumor miRs or siRNAs, have been found to be efficiently encapsulated into MSC-Exo by the transfection approach [110, 111, 115]. Han and colleagues studied exosomes from human cord blood-derived MSCs as a targeted delivery system for AMOs in CRC therapy [104]. They employed electroporation to load anti-miR-122 (miravirsen) into the exosomes decorated by iRGD peptides that endow exosomes with highly efficient tissue penetration abilities and a specific binding ability to tumor cells [104]. The iRGD peptide accelerates drug delivery into tumor cells. As a ligand, the iRGD peptide can target tumor cells expressing high levels of the neuropilin-1 (NRP-1) receptor, mediates the penetration into the cell membrane, and efficiently kills tumor cells [109, 114, 116]. Of note, NRP-1 was identified to be strongly expressed in colon cancer cell lineages and tissues, permitting the iRGD-Exo-antimiR-122 to be effectively ingested by colon cancer cells and significantly inhibiting tumor growth in vitro and in vivo [104]. Notably, iRGD-Exo-antimiR-122 was shown to be highly enriched in tumor sites and exerted excellent anti-tumor efficacy in a xenograft CRC model [104].

There is also another study that used the modified BM-MSC-Exo as a carrier for doxorubicin (Dox) in targeted therapy of CRC in an animal model. Of note, Dox-loaded BM-MSC-Exo were decorated with the carboxylic acid-end MUC1 aptamer (5TR1) to provide selective guided drug delivery into CRC cells. The 5TR1-BM-MSC-Exo could highly enhance the anti-tumor activity of Dox against CRC both in vitro and in vivo while decreasing its toxicity on non-targeted cells and tissues. The targeted Dox-BM-MSC-Exo showed higher tumor accumulation and faster liver clearance in comparison with free DOX, suggesting it as a valuable platform for the safe and versatile delivery of DOX to CRC in the clinical application [117].

## Concluding remarks, challenges, and future perspectives

An overall conclusion from the above preclinical findings indicates that MSC-Exo can provide a novel therapeutic approach in treating UC and CRC. Although both UC and CRC have an inflammatory pathological basement and UC is the main risk factor for CRC development, MSC-Exo can exert the protective role against UC and CRC through two different mechanisms including modulating immune responses and inducing anti-tumor responses, respectively. Of note, exosomes as a cell-free delivery system have advantages over the corresponding stem cells: exosomes are smaller and less complex than stem cells and show the potential to circumvent some shortcomings of stem cells, such as the need for a persistent administration of cells with stable phenotype, tumorigenicity and immune rejection, ectopic tissue formation, and the infusional toxicity caused by transplanted cells.

Of note, hUC-MSC-Exo have been found to ameliorate the experimental UC through inhibiting infiltration and function of inflammatory macrophages, suppressing the differentiation of inflammatory Th17 cells, reducing the expression of proinflammatory cytokines, as well as inducing the differentiation of anti-inflammatory Th2 cells and enhancing the expression of anti-inflammatory cytokines. However, the anti-tumor activity of hUC-MSC-Exo on CRC cells is attributed to the existence of tumor-suppressive miR-3940-5p that can suppress the growth, invasion, and metastasis of CRC through downregulating the expression of oncoprotein ITGA6.

Moreover, it has been found that IFN-γ-primed BM-MSC-Exo contain high levels of miR-125a and miR-125b that can ameliorate colitis phenotypes through inhibiting differentiation of Th17 cells and promoting the proliferation of Treg cells via downregulating the Stat3 expression. Notably, BM-MSC-Exo containing tumor-suppressive miRs, including miR-22-3p and miR‐16‐5p, have been shown to suppress proliferation, migration, and invasion of CRC cells via regulation of RAP2B/PI3K/AKT signaling pathway and ITGA2. Thus, BM-MSC-Exo containing miR-125a/miR-125b or miR-22-3p/miR‐16‐5p have the valuable potential as therapeutic tools for treating UC or CRC, respectively. Besides, there is also a report that shows BM-MSC-Exo can induce CRC development through transporting tumorigenic miR-424 into colon cells, suggesting Exo-miR-424 as a promising therapeutic target whose inhibition may provide a novel strategy in CRC treatment.

On the other hand, there have also been reports that show the therapeutic potential of exosomes derived from other sources of MSCs, including AD-MSCs and OE-MSCs, in UC treatment; however, there is no report regarding their effect on CRC. It was shown that AD-MSC-Exo could attenuate typical symptoms of UC, decrease histological scores of DAI, improve colonic ulcers, decrease the infiltration of inflammatory cells and production of proinflammatory cytokines, and increase percentages of anti-inflammatory Treg cells and the anti-inflammatory cytokines in mice with acute colitis. OE-MSC-Exo could significantly ameliorate the severity of UC through immunomodulatory activity by suppressing Th1/Th17 cell pro-inflammatory responses and enhancing Treg cell anti-inflammatory responses. Thus, in addition to hUC-MSC-Exo and BM-MSC-Exo, the exosome therapy by AD-MSC-Exo and/or OE-MSC-Exo can donate a novel approach for UC treatment.

Of note, the safety and therapeutic efficiency of exosomes have been reported in preliminary clinical trials for treating CRC [118]; however, engineering of delivery properties can improve the therapeutic efficiency of exosomes in further clinical use. One of the main issues with exosome-based delivery systems is rapid clearance by the reticuloendothelial system (RES). Notably, the surface modification using “don’t eat me” signaling molecules like CD47 and PEG can reduce uptake by RES. Another highly attractive feature of exosomes as a drug delivery vehicle is their intrinsic homing capability to target tissues. Of note, decorating the exosome surface via specific ligands, such as the iRGD peptide or the carboxylic acid-end MUC1 aptamer, can elevate the local concentration of exosomes at the target disease cells or tissues, thereby minimizing side effects and the toxicity and maximizing therapeutic efficiency [104].

Moreover, there are still several drawbacks that limit the clinical application of exosome therapy in UC or CRC. Current challenges include the following: the technology of large-scale culture and isolation of MSCs; the optimal method for the long-term preservation of exosomes; the efficient and cost–benefit techniques to rapidly isolate, purify, quantitate, and identify exosomes; transplantation conditions of exosomes; as well as their cost. Thus, to facilitate the clinical use of exosome therapy in UC or CRC, more in-depth analysis for circumventing such problems is necessary.

## Data Availability

Not applicable.
